# Oxidative stress in skin fibroblasts cultures from patients with Parkinson's disease

**DOI:** 10.1186/1471-2377-10-95

**Published:** 2010-10-19

**Authors:** Pilar del Hoyo, Alberto García-Redondo, Fernando de Bustos, José Antonio Molina, Youssef Sayed, Hortensia Alonso-Navarro, Luis Caballero, Joaquín Arenas, José AG Agúndez, Félix Javier Jiménez-Jiménez

**Affiliations:** 1 Departamento de Bioquímica - Investigación, Hospital Universitario Doce de Octubre, Madrid, Spain; 2 Servicio de Bioquímica, Hospital Nuestra Señora del Prado, Talavera de la Reina, Toledo, Spain; 3 Servicio de Neurología, Hospital Universitario Doce de Octubre, Madrid, Spain; 4 Departamento de Medicina-Neurología. Hospital. "Príncipe de Asturias", Universidad de Alcalá, Alcalá de Henares, Madrid, Spain; 5 Sección de Neurología, Hospital La Mancha-Centro, Alcázar de San Juan, Ciudad Real, Spain; 6 Departamento de Farmacología y Psiquiatría, Universidad de Extremadura, Badajoz, Spain; 7 Sección de Neurología, Hospital del Sureste, Arganda del Rey, Madrid, Spain

## Abstract

**Background:**

In the substantia nigra of Parkinson's disease (PD) patients, increased lipid peroxidation, decreased activities of the mitochondrial complex I of the respiratory chain, catalase and glutathione-peroxidase, and decreased levels of reduced glutathione have been reported. These observations suggest that oxidative stress and mitochondrial dysfunction play a role in the neurodegeneration in PD. We assessed enzymatic activities of respiratory chain and other enzymes involved in oxidative processes in skin fibroblasts cultures of patients with PD.

**Methods:**

We studied respiratory chain enzyme activities, activities of total, Cu/Zn- and Mn-superoxide-dismutase, gluthatione-peroxidase and catalase, and coenzyme Q10 levels in skin fibroblasts cultures from 20 Parkinson's disease (PD) patients and 19 age- and sex- matched healthy controls.

**Results:**

When compared with controls, PD patients showed significantly lower specific activities for complex V (both corrected by citrate synthase activity and protein concentrations). Oxidized, reduced and total coenzyme Q10 levels (both corrected by citrate synthase and protein concentrations), and activities of total, Cu/Zn- and Mn-superoxide-dismutase, gluthatione-peroxidase and catalase, did not differ significantly between PD-patients and control groups. Values for enzyme activities in the PD group did not correlate with age at onset, duration, scores of the Unified Parkinson's Disease Rating scales and Hoehn-Yahr staging.

**Conclusions:**

The main result of this study was the decreased activity of complex V in PD patients. This complex synthesizes ATP from ADP using an electrochemical gradient generated by complexes I-IV. These results suggest decreased energetic metabolism in fibroblasts of patients with PD.

## Background

The pathogenesis of the neuronal degeneration of neurons in the pars compacta of the substantia nigra in patients with Parkinson's disease (PD) is unknown. The discovery that the 1-methyl-4-phenyl-pyridinium ion (MPP^+^), the metabolite of the parkinsonism-inducing neurotoxin 1-methyl-4-phenyl-1,2,3,6-tetrahydropyridine (MPTP) did inhibit complex I [1-3] led some investigators to examine the mitochondrial function in idiopathic PD.

The mitochondrial respiratory chain consists of five enzymatic complexes located within the inner mitochondrial membrane. Four enzymes (complexes I-IV) transport electrons from NADH or succinate to oxygen, and these complexes pump protons out of the mitochondria to form an electrochemical gradient. Complex V (ATP synthase) uses that electrochemical gradient to synthesize ATP from ADP.

Many studies have shown decreased complex I activity in the substantia nigra of PD patients (reviewed in reference [4]). More recently, Parker et al. [5] found decreased complex I activity in frontal cortex as well. However, the results of studies measuring mitochondrial complexes in peripheral tissues have been controversial (reviewed in reference [4]). The activity of mitochondrial complexes has been measured in the following tisues:

• Skeletal muscle (most studies performed with isolated mitochondria). There have been reported normal activities of mitochondrial respiratory chain complexes I-IV [6-10], or decreased activities of complexes I [11-16], II [11-14] or IV [15-17].

• Platelets (most studies performed with isolated mitochondria). There have been reported normal activities [6,18-20], or decreased activities of complex I [21-26], complex II+III [25] or complex IV [24].

• Lymphocytes. Studies with homogenates showed decreased activities of complex II [23] or complexes I and IV [27], while studies with isolated mitochondria showed normal activities [28], or decreased activity of complexes I and I+III [29], and complex IV [29].

• Leukocytes: Muftuoglu et al. [30] reported decreased complexes I and IV activities in leukocytes from patients with idiopathic PD, and decreased complex I activity, with normality of complex IV, in patients with parkin mutations.

• Spermatozoa. Our group showed similar mitochondrial respiratory chain enzymes activities corrected by citrate synthase (CS), in the spermatozoa from untreated PD patients [31].

A study on mitochondrial function of skin fibroblasts from PD showed a deficiency in complexes I and IV [32,33] that was partially restored with coenzyme Q_10 _treatment [33]. Piccoli et al. [34] examined mitochondrial respiratory function of a patient affected by early-onset parkinsonism carrying the homozygous W437X nonsense mutation in the PINK1 gene, and found decreased complex IV activity and some depression of the ATPase activity. In a recent study, Ferrer et al. [35] have shown decreased levels of ATP synthase in the substantia nigra and increased levels in the frontal cortex of patients with PD.

The study of mitochondrial function in PD might be important because, together with the classical description of oxidative stress and mitochondrial dysfunction in the brains of patients with this disease, the identification of specific gene mutations that cause PD has reinforced their relevance. Proteins associated with familial PD, such as PTEN-induced putative kinase 1 (PINK1), DJ-1, alpha-synuclein, leucine-rich repeat kinase 2 (LRRK2) and, possibly, parkin, are either mitochondrial proteins or are associated with mitochondria, and have a role on oxidative stress [36].

Some authors described decreased activities of the antioxidant enzymes glutathione-peroxidase (GPx) [37,38], total superoxide-dismutase (SOD) [39], Mn-SOD [40], Cu,Zn-SOD [41], and catalase [37], in the substantia nigra of PD patients; while Mn-SOD activity in the cortex of PD patients has been found increased [35,42]. GPx activity has been studied in serum or plasma, erythrocytes, and neutrophils; total SOD, Cu,Zn-SOD and/or Mn-SOD levels or activities in serum or plasma, cerebrospinal fluid, erythrocytes, neutrophils, platelets, and lymphocytes; and catalase activity in erythrocytes from PD patients. The results of these studies (reviewed in reference [4]) are controversial. Piccoli et al. [34], in their patient with the homozygous W437X nonsense mutation in the PINK1 gene described normal levels of total glutathione, reduced glutathione and glutathione peroxidase activity, normal Cu/Zn-SOD activity and a small decrease of the mitochondrial Mn-SOD in fibroblasts. To our knowledge activity of catalase has not been studied previously in fibroblasts.

Coenzyme Q_10 _(CoQ_10_) is the electron acceptor for mitochondrial complexes I and II and a powerful antioxidant [43]. Shults et al. [44] reported a correlation between mitochondrial CoQ_10 _levels and activities of complexes I and II/III. Gotz et al. [45] reported a decreased [reduced CoQ10/oxidized CoQ10] ratio (redox ratio) in untreated PD patients, that was further decreased by levodopa treatment and partially restored with selegiline. Serum or plasma CoQ_10 _levels have been described decreased, normal or increased in some studies (reviewed in reference [4]). To our knowledge, CoQ_10 _levels in fibroblasts of PD patients have not been measured previosly.

The aim of this study was to assess the enzymatic activities of respiratory chain enzymes and other enzymes involved in oxidative processes, such as GPx, SOD, catalase and coenzyme Q_10 _in skin fibroblast cultures from patients with PD. The study was carried out in skin fibroblasts because the specimens were easily accessible and should be free of influence from medication, environmental hazards and other possible factors contributing to oxidative stress.

## Methods

### Patients and controls

Twenty patients diagnosed with PD and 19 healthy age- and sex-matched controls were enrolled in this study, after informed consent. The study was approved by the Ethics Committees of the University Hospitals "Doce de Octubre" and "Príncipe de Asturias", and was conducted according to the declaration of Helsinki. Two patients received no treatment, while the other 18 were treated with antiparkinsonian drugs alone or in combination including levodopa (16 cases), dopamine agonists (14 cases), selegiline or rasagiline (8 cases), anticholinergics (3 cases), and amantadine (1 case).

The control group was composed by 19 patients evaluated in the neurology departments of the same hospitals because of tension type headache, dizziness, dorsolumbar or cervical pain, etc. The clinical data of PD patient and control groups are summarized in table 1.

**Table 1:** Clinical data of Parkinson's disease (PD) and control patients groups.

*VARIABLE*	*PARKINSON'S DISEASE (n = 20)*	*CONTROLS (n = 19)*	*p values*
MEAN (SD) AGE (years)	59.2(15.6)	59.16 (10.7)	n.s
SEX (F/M)	8/12	13/7	
MEAN (SD) AGE AT ONSET OF PD (years)	51.1(10.2)		
MEAN (SD) DURATION OF PD (years)	8.1(4.2)		

The following exclusion criteria were applied both to patients and controls: A) Ethanol intake higher than 80 g/day in the last 6 months. B) Previous history of chronic hepatopathy or diseases causing malabsorption. C) Previous history of severe systemic disease. D) Atypical dietary habits (diets constituted exclusively by one type of foodstuff, such as vegetables, fruits, meat, or others, special diets because of religious reasons, etc) F) Intake of drugs which modify lipid absorption. G) Therapy with vitamin supplements in the last 6 months.

### Skin fibroblast cultures

Human skin fibroblasts were obtained from the dorsal region of the upper arm of each PD patient or control. Fibroblasts from the biopsy specimens were cultured in Dulbecco's modified Eagle's medium containing penicillin (100 UI/mL), streptomycin (100 mg/dl), L-glutamine (4 mM) and supplemented with heat-inactivated fetal calf serum at 37°C in a humidified atmosphere of 95% air and 5% CO_2_. Cells were grown to confluence, harvested by trypsinization at 37°C, washed with culture medium and resuspended with phosphate buffer 20 mM, and then sonicated to obtain the cell homogenate. Care was taken not to use cultures with a passage number greater than 12.

### Respiratory chain enzymes assay

Respiratory chain enzymes and citrate synthase activity were measured in a DU-68 spectrophotometer (Beckman), applying 35-150 μg of total protein per 1 ml test volume in every complex enzyme assay. Incubation temperatures were 30°C for NADH coenzyme Q oxidoreductase (complex I), rotenone-sensitive NADH cytochrome c reductase (complexes I and III), succinate cytochrome c reductase (complexes II and III), succinate dehydrogenase (complex II) and citrate synthase (CS), and 38°C for cytochrome c oxidase (complex IV). The activities of complexes II, I and III, II and III, IV, and CS were determined as reported elsewhere [27]. Complex I was measured by following the oxidation of NADH at 340 nm in 100 mM Tris-HCl pH 7.4, 500 mM sucrose, 2 mM EDTA, 5 mM KCN, 100 μM NADH, and 50 μM DB (2,3-dimethyl-5-decyl-6-methylbenzoquinone) [46]. The reaction was inhibited by 90% when 2 μM rotenone was present. Complex V was determined by measuring 2.5 mM ATP extinction in a mean with 50 mM Hepes-Mg buffer at pH 8.0, 0.2 mM NADH, and phosphoenol-pyruvate 2.5 mM, and then adding 5 μl of pyruvate-kinase (10 mg/ml) and 10 μL of lactate-dehydrogenase (5 mg/ml) in presence of 10 μl of antimycin A (0.2 mg/ml in 50% ethanol). The oligomycin sensitive fraction was measured by adding 10 μl of oligomycin (0.2 mg/ml in 50% ethanol).To correct for mitochondrial volume, all respiratory chain enzyme activities were normalized to the activity of CS. Protein were measured by the method of Lowry et al. [47]. Specific activities were expressed as nmol × min^-1 ^xmg protein^-1^, and referred to the specific activities of CS to correct for mitochondrial volume. All chemicals were from Boehringer Mannheim (Boehringer Mannheim, Germany) and Sigma Chemicals (St. Louis, MO). The measures were performed three times for every sample on three different passages (most of them between passage 5 and passage 10). The standard deviation of every measure is specified in table 2. The range of linearity was defined in all our determinations in a wide range of protein concentration. So we decided to use the homogenates in order to have a total protein concentration of 35-150 micrograms per mililiter of final test volume. This range was inside the linearity to all the activities of the OXPHOS complexes.

**Table 2:** Mean (SD) respiratory chain enzymes activities (expressed as nmol/min/mg protein) in skin fibroblast cultures of patients with Parkinson's disease (PD) and controls (CS = citrate synthase).

*VARIABLE*	*PARKINSON'S DISEASE (n = 20)*	*CONTROLS (n = 19)*	*p values*
COMPLEX I/CS	34.56 (17.10)	26.13 (9.49)	n.s.
COMPLEX II/CS	19.02 (3.22)	17.10 (2.77)	n.s.
SDH/CS	14.45 (2.83)	12.71(3.35)	n.s
COMPLEX III/CS	26.80 (7.97)	26.75 (5.86)	n.s.
COMPLEX IV/CS	65.70 (10.83)	62.01 (9.61)	n.s.
COMPLEX V/CS	41.57 (16.70)	52.52 (23.56)	0.021
COMPLEX I + III/CS	380.70 (88.90)	337.91 (58.85)	n.s.
COMPLEX II + III/CS	16.67 (5.03)	12.27 (3.33)	n.s
CS/protein	77.39 (28.59)	74.27 (20.08)	n.s
COMPLEX I/protein	26.70 (11.13)	22.48 (9.96)	n.s.
COMPLEX II/protein	13.73 (3.79)	13.22 (3.70)	n.s.
SDH/protein	10.07 (2.85)	10.17 (3.70)	n.s.
COMPLEX III/protein	20.82 (9.20)	20.10 (7.51)	n.s.
COMPLEX IV/protein	44.49 (10.53)	44.49 (11.42)	n.s.
COMPLEX V/protein	29.95 (14.70)	42.21 (18.80)	0.017
COMPLEX I + III/protein	253.76 (79.91)	247.25 (51.31)	n.s.
COMPLEX II + III/protein	9.89 (3.11)	9.48 (4.59)	n.s.

### Glutathione-peroxidase, catalase and superoxide-dismutase isoenzymes determination

Glutathione peroxidase specific activity was determined according to the method described by Flohé and Günzler [48] based on NADPH oxidation followed at 340 nm at 37°C.

Catalase activity was determined according to the method described by Aeby [49] based on H_2_O_2 _decomposition followed at 240 nm at room temperature. Catalase specific activity was determined by calculating the rate constant of a first order reaction.

Total and Mn-SOD activities were determined according to the method described by Spitz and Oberley [50] based on nitroblue tetrazolium reduction by superoxide radicals followed at 560 nm at room temperature. The Mn-SOD was distinguished from cyanide-sensitive Cu/Zn-SOD by the addition of 5 mM NaCN. Cu/Zn-SOD activity was calculated by substracting the cyanide-resistant SOD activity form the total SOD activity. One unit of SOD activity is defined as the amount of enzyme that inhibits the reaction rate by 50%.

All enzymatic activities were expressed as values normalized to total cellular protein. The measurements were performed three times for every sample.

### Coenzyme Q_10 _determinations

Oxidized, reduced and total coenzyme Q_10 _levels were determined by high performance liquid chromatography with electrochemical detection. The method used was that of Langedijk et al. [51] with some modifications. The stationary phase was a reverse phase column (HR-80 RP-C_18_, 80 × 4,6 mm. ESA Inc). The mobile phase was prepared by dissolving 7 g of NaClO_4_.H_2_O in 1000 ml of methanol/propanol/HClO_4 _70%, 700.8:200:0.2 (vol/vol), and the flow rate was set at 0.8 ml/min. The programmed conditions for the electrochemical detector and the post-column valve were the same. The system was entirely controlled by a computer (Kromasystem 2000, Kontron Instruments). Injections were made in a 50 μl injection valve (Model 7161, Rheodyne, Cotaty, USA) with a 100 μl syringe from Hamilton (Bonaduz, Switzerland). The calibration method used ubiquinone as external standard. The within-run coefficients of variation for CoQ_10 _and CoQH_2 _were, respectively, 5 and 3.2%, and the day to day precisions were 9.2 and 6.3%. CoQ_10 _recovery ranged between 88 and 93%. The measurements of CoQ_10 _were expressed in nmol/g of protein. The measures were performed three times for every sample.

### Statistical analysis

Results were expressed as mean ± SD. Statistical analysis was done by the SPSS statistical package (15.0 version). For continuous variables the Kolmogorov-Smirnoff test was used to analyze normality in the distribution. Then, the Students's two-sample t-test was used for variables that followed a normal distribution and the Mann-Whitney test was used for the rest of variables. The results of each table were corrected for multiple comparisons by the use of Bonferroni's correction. Correlation analyses were performed by using Pearson's correlation coefficient.

## Results

**Figure 1:**
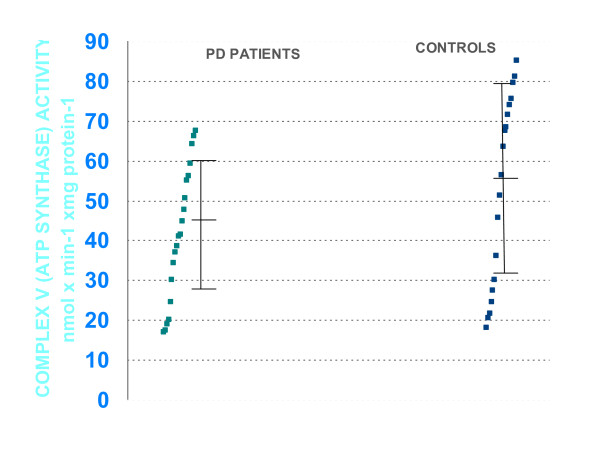
**Values of complex V (ATP-synthase) activity in Parkinson's disease patients and in controls (measured as mmol/min/mg of protein, corrected for citrate synthase)**.

The results are summarized in tables 2 and 3. When compared with controls, PD patients showed significantly lower specific activities for complex V (both corrected by citrate synthase activity and protein concentrations, table 2, figure 1). Oxidized, reduced and total coenzyme Q10 levels (both corrected by citrate synthase and protein concentrations), and activities of total, Cu/Zn- and Mn-superoxide-dismutase, gluthatione-peroxidase and catalase, did not differ significantly between PD-patients and control groups (table 3). Values for enzyme activities in the PD group did not correlate with age at onset, duration, scores of the Unified Parkinson's Disease Rating scales and Hoehn-Yahr staging.

**Table 3:** Mean (SD) superoxide-dismutase (SOD), glutathione-peroxidase (GPx), and catalase activities (expressed as units/mg protein); and concentrations of coenzyme Q_10 _(expressed in nmol/g protein) in skin fibroblast cultures of patients with Parkinson's disease (PD) and controls (CS = citrate synthase).

*VARIABLE*	*PARKINSON'S DISEASE(n = 20)*	*CONTROLS (n = 19)*	*p values*
REDUCED CoQ10/CS	0.41 (0.16)	0.34 (0.11)	n.s.
OXIDIZED CoQ10/CS	0.75 (0.26)	0.63 (0.23)	n.s.
TOTAL CoQ10/CS	1.16 (0.33)	0.97 (0.25)	n.s.
REDUCED CoQ10/protein	24.50 (7.38)	24.50 (7.38)	n.s.
OXIDIZED CoQ10/protein	56.49 (25.20)	47.31 (23.50)	n.s.
TOTAL CoQ10/protein	86.27 (29.07)	71.86 (26.38)	n.s.
OXIDIZED Q10/Reduced Q10	0.60 (0.27)	0.62 (0.27)	n.s.
MnSOD/protein	14.20 (8.84)	11.76 (7.88)	n.s.
CuZnSOD/protein	8.80 (7.08)	8.90 (6.87)	n.s.
TOTAL SOD/protein	26.00 (8.58)	20.72 (11.52)	n.s.
GPx/protein	13.03 (4.96)	14.45 (6.47)	n.s.
CATALASE/protein	9.71 (5.20)	7.26 (2.05)	n.s.

## Discussion

The results of the present study showed that in PD patients there was a decreased activity of mitochondrial respiratory chain complex V, corrected by CS activity, in skin fibroblast cultures. The results on complexes I and IV in this study did not differ significantly between PD patients and control groups, and none of our patients showed decreased activities of these complexes. These results contrast with those reported by other group [32,33], who found a deficiency in complexes I and IV activities, specially in a subgroup of PD patients compared with controls, using skin fibroblast cultures as well. This deficiency was partially restored with coenzyme Q_10 _treatment [33].

The discrepancies between the results on complex I and IV of the present study and those of Winkler-Stuck et al. [32,33] could be related with several methodological reasons. These authors found enhanced flux control coefficient of complex I and IV. This kind of variation was measured using inhibitor titrations and calculating the flux control coefficients from titration curves. The method used by Winkler-Stuck et al. [32,33] is different from that used in our study, because we only have measured, in the case of mitochondrial respiratory chain complex activities, the activity of every complex, and not the flux control coefficient. In addition, the control groups were different, because while Winkler-Stuck et al. [32,33] included patients who performed muscle biopsy because they had discrete myopathic EMG abnormalities but withoutt biopsy evidence of myopathy, while our control patients had no clinical symptoms or signs suggesting myopathy. Winkler-Stuck et al. [32,33] did not measure complex V activity.

Our results suggest that decreased complex I activity seen in the substantia nigra of PD patients is not a generalised phenomenon. It is of note that complex V has not been usually studied neither in brain nor in peripheral tissues from PD patients. Indeed, complex V was not measured in previous studies by our group using isolated mitochondria from lymphocytes [27] or spermatozoa [31] or in other report using skin fibroblasts [32,33]. Cardellach et al. [15] found decreased complex V activity in muscle of 2 out of their 8 parkinsonian patients and, more recently, Ferrer et al. [35] found decreased levels of ATP-synthase (complex V) in the substantia nigra and increased levels of this enzymatic complex in the frontal cortex of patients with PD.

The activities of Cu/Zn- and Mn-SOD, GPx, and catalase, and the oxidized, reduced, and total CoQ_10 _levels in skin fibroblasts of PD patients were similar to those of controls. To our knowledge, these values have not been previously measured in this tissue form, with the exception of the single patient with a mutation in the PINK1 gene reported by Piccoli et al. [34]. These results do not rule out the possibility that there may be regional deficiencies of the activities of these enzymes and of CoQ_10 _concentrations in some areas of the brain. Moreover, none of the enzymatic activities measured was correlated with the analyzed clinical features of PD.

## Conclusions

The main result of the present study in skin fibroblasts cultures suggests a possible contribution of the deficiency of complex V activity, but not of other enzymes related with oxidative stress, to the pathogenesis of PD. This result is in agreement with that reported by Ferrer et al [35] in the PD substantia nigra, which was interpreted by the authors as related with neuronal loss. Complex V is involved in the synthesis of ATP from ADP, and is very important in the energetic metabolism of the cells. The result of the present study suggests decreased energetic metabolism in fibroblasts of patients with PD.

## Competing interests

The authors declare that they have no competing interests.

## Authors' contributions

(all authors read and approved the final manuscript)

• PDH participated in the conception and design of the study, in the obtention of fibroblast cultures, biochemical analysis, drafting and critical revision of the manuscript, and supervision.

• AGR participated in the conception and design of the study, in the obtention of fibroblast cultures, biochemical analysis, drafting and critical revision of the manuscript, and supervision.

• FDB participated in the conception and design of the study, in the obtention of fibroblast cultures, biochemical analysis, drafting and critical revision of the manuscript, and supervision.

• JAM participated in the conception and design of the study, in the recruitment and clinical evaluation of patients and controls, drafting and critical revision of the manuscript, obtaining funding, and supervision.

• YS participated in the conception and design of the study, in the recruitment and clinical evaluation of patients and controls, and drafting and critical revision of the manuscript.

• HAN participated in the conception and design of the study, in the recruitment and clinical evaluation of patients and controls, and drafting and critical revision of the manuscript.

• LC participated in the conception and design or the study, and drafting and critical revision of the manuscript.

• JA participated in the conception and design or the study, drafting and critical revision of the manuscript, and supervision.

• JAGA participated in the conception and design of the study, performed statistical analysis, participated in the drafting and critical revision of the manuscript, and supervision.

• FJJJ participated in the conception and design of the study, in the recruitment and clinical evaluation of patients and controls, drafting and critical revision of the manuscript, obtaining funding, and supervision.

## Pre-publication history

The pre-publication history for this paper can be accessed here:

http://www.biomedcentral.com/1471-2377/10/95/prepub
